# Correlations between receptor occupancy change and mental state in patients using long-acting injectable antipsychotics: MIDILIA pilot study

**DOI:** 10.1192/bjo.2025.10831

**Published:** 2025-09-12

**Authors:** James R. O’Neill, Christopher Wilson, Mark A. Horowitz, Samantha L. McLean, Muhammad Faisal, Michael Dixon, Lewis Couchman, Katie Lawlor, George Crowther

**Affiliations:** School of Medicine, University of Leeds, UK; Leeds and York Partnership NHS Foundation Trust, Leeds, UK; South West Yorkshire Partnership NHS Foundation Trust, Wakefield, UK; Institute of Psychiatry, Psychology & Neuroscience, King’s College London, UK; North East London Foundation Trust, London, UK; School of Pharmacy and Medical Sciences, University of Bradford, UK; Wolfson Centre for Applied Health Research, Bradford, UK; Faculty of Health Studies, University of Bradford, UK; NIHR Yorkshire & Humber Patient Safety Research Collaboration (PSRC), Bradford, UK; Analytical, Environmental and Forensic Sciences, King’s College, London, UK; Analytical Services International, London, UK

**Keywords:** Psychosis, long-acting injectable, antipsychotic, psychopharmacology, pharmacokinetic

## Abstract

**Background:**

The rate at which psychosis drugs can be reduced in dose remains unclear. Anecdotal reports exist of people experiencing worsening of mental state before their next dose of long-acting injectable antipsychotic. No research has previously explored this phenomenon, but understanding this may advise on the rate of receptor occupancy change that provokes the emergence of psychotic symptoms.

**Aims:**

Exploring the relationship between psychotic symptoms and variations in plasma concentration (and calculated receptor occupancy) of long-acting injectable antipsychotics.

**Method:**

This longitudinal study monitored mental state variation within dosing cycles of people taking depot flupentixol and zuclopenthixol. The Positive and Negative Syndrome Scale (PANSS) monitored global mental state changes, and was stratified into domains according to a five-factor model. Plasma assays at maximal and minimal concentrations allowed prediction of striatal D_2_ occupancy from published data. We examined correlations between receptor occupancy and the emergence of psychotic symptoms.

**Results:**

Preliminary results from ten participants with psychotic disorders suggest that global mental state deterioration may correlate with increased rate of D_2_ occupancy reduction. Increased rate of D_2_ occupancy reduction led to deterioration in ‘positive’ (*r* = 0.637 [CI: 0.013, 0.904], *P* = 0.047) and ‘resistance’ (*r* = 0.726 [CI: 0.177, 0.930], *P* = 0.018) PANSS clinical domains at minimal concentrations. PANSS score differences were not related to absolute reduction in D_2_ occupancy.

**Conclusions:**

Our novel observational study design has been demonstrated to be feasible and practicable. Faster reductions in D_2_ occupancy may increase the risk of increased positive psychotic symptoms and irritability. Slower reductions may minimise this effect. Further recruitment is required before this can be confirmed.

Medications known as ‘antipsychotics’ can be effective in treating acute psychotic illnesses.^1,2^ However, these often result in debilitating^3,4^ and dose-dependent^5,6^ side-effects that can affect a person’s quality of life.^7^ Establishing the optimal dose for such medications, and whether the medication is still warranted, is therefore essential for clinicians.

## Long-acting injectable antipsychotics

Long-acting injectable antipsychotics (LIAs), also known as ‘depots’, have prolonged elimination half-lives^8,9^ which result in less rapid disruption of neuroreceptor activity, including at dopamine D_2_ receptors. It has previously been postulated that this prolonged action can sometimes allow LIAs to be stopped abruptly because the medication would ‘self-taper’. However, in silico modelling has suggested that the peak rate of D_2_ receptor occupancy change (RODOC) resulting from abrupt cessation of LIAs may still be excessively rapid, thus increasing the risk of symptomatic relapse.^10,11^

## Inter-dose variation

Drug concentrations of LIAs fluctuate by up to fourfold between injections.^8^ It has been anecdotally reported that some people develop increased psychotic symptoms as they approach the due date for their next dose of LIA. This is presumably consistent with an inter-dose withdrawal effect, as observed with significant reductions or abrupt discontinuation of antipsychotics.^12^ We are not aware of any formal investigation of inter-dose withdrawal for LIAs documented in the academic literature.

## Interpersonal variation

Different LIAs have varying half-lives due to varying mechanisms of action, metabolism and elimination.^13^ In addition, interpersonal metabolism of psychosis drugs varies substantially.^9^ Exploring the effects of variation in drug concentration, and subsequently RODOC, therefore provides a natural experiment allowing examination of the relationship between RODOC and mental state changes. This may shed light on a rate of dose reduction for psychosis drugs that does not provoke worsening of mental state and is therefore tolerated by patients.^14^

## Hypothesis

We hypothesise that faster RODOC during the inter-dose interval^10,11^ (produced by LIAs at lower doses or with shorter elimination half-lives) would lead to more prominent changes in mental state than slower RODOC (produced by LIAs with longer half-lives or prescribed at higher doses).^10,11^

## Aims

We aimed to assess whether, and when, symptoms of antipsychotic withdrawal (manifesting as worsened mental state) emerge during the interval between administrations of LIAs. Our secondary aim was to determine the acceptable and tolerable rate of change in drug concentration and D_2_ occupancy for patients prescribed LIAs.

## Method

### Design

The ‘Monitoring the Inter-Dose Interval of Long-acting Injectable Antipsychotics’ (MIDILIA) study was a longitudinal observational study that monitored mental state and plasma concentrations (with extrapolation to receptor occupancy) at different points during a participant’s LIA dosing cycle. No interventional changes to a participant’s existing treatment were made during the study.

### Schedule of events

Two sampling sessions took place with each participant, occurring as close as possible to the anticipated points of maximal and minimal drug concentration^8,9^ during the same inter-dose interval. At each session, a Positive and Negative Syndrome Scale (PANSS) semi-structured interview^15^ was completed and a serum drug assay obtained from each participant. Plasma drug concentrations were used to account for interpersonal differences in metabolism, and subsequently to predict the expected striatal D_2_ occupancy^4,16^ at each interview.

### Setting

Sampling sessions were completed in either an in- or out-patient setting depending on current clinical status, but the setting was kept as consistent as possible across both sessions to reduce confounding factors. This included time of day, day of the week, environment and any other personnel (e.g. staff, family or friends) present at each meeting, in addition to the participant and researcher.

### Ethical approval

The authors assert that all procedures contributing to this work comply with the ethical standards of the relevant national and institutional committees on human experimentation, and with the Helsinki Declaration of 1975 as revised in 2013. All procedures involving human participants were approved by the Health Research Authority of England and Wales (Research Ethics Committee, ref. 23/SC/0156). This granted ethical approval for the research team to recruit participants from a single National Health Service (NHS) mental health trust receiving either LIA zuclopenthixol or flupentixol.

### Inclusion and exclusion criteria

MIDILIA recruited adults (18–80 years) who had received at least five consecutive LIA administrations of a consistent drug, dose and dosing interval. The study excluded anyone with significant hepatic or renal impairment, anyone on concurrent oral antipsychotic medication and anyone deemed not yet to have reached a steady-state plasma concentration of their LIA medication (i.e. had received fewer than five consistent administrations of their current drug and dose).

### Recruitment

Participants were identified using existing clinical networks within an NHS mental health trust, with the direct clinical team referring potential participants to the MIDILIA research team. All participants were deemed able to give capacitous informed consent to participate within the study, and written consent was obtained from all participants.

### Timing of sessions

Expected timings for maximal drug concentrations were determined by derived *t*_max_ values from existing pharmacokinetic literature^8,9^ for individual drugs. Both flupentixol and zuclopenthixol decanoate reach maximal concentrations between 4 and 7 days after injection. The sampling session relating to minimal drug concentration took place within 24 h of the following LIA administration being due.

### Assessment of mental state

Participant mental state at each sampling session was assessed through the completion of PANSS interviews^15^ with participants at each session. PANSS has been validated and is widely used in mental health research as a tool to assess the symptom severity of psychosis.^17^ Global PANSS scores were used to account for the broad and wide-ranging syndrome by which antipsychotic withdrawal may manifest.^18^ However, stratification of PANSS domains as separate outcome variables was also undertaken using the five-factor model of PANSS.^19^ These comprised positive symptoms, negative symptoms, cognitive disorganisation, affective domains and resistance domains. The resistance domain comprised rater scores for hostility, impulse control, excitement and uncooperativeness.^19^

Two researchers (J.R.O. and C.W.) were involved in conducting PANSS interviews with participants. An interrater reliability check was conducted of global PANSS scoring using three video case examples prior to commencing the study. The intra-class coefficient (ICC)^20^ demonstrated reliability between the two raters to be moderate to good (ICC = 0.691, 95% CI [0.551, 0.786]).

### Drug concentration analysis

All participants provided at least one, but preferably two, blood samples during their inter-dose interval for determination of individualised drug concentrations. Samples were obtained through venepuncture and mixed with ethylenediaminetetraacetic acid (EDTA), before being sent immediately for centrifugation, which occurred within 24 h. Plasma was harvested and samples were then frozen until time of analysis, which was within 3 months for zuclopenthixol^21,22^ and within 12 months for flupentixol,^23^ based on previously published stability data. Analysis was performed by liquid chromatography with mass spectrometric detection, using certified reference materials from two independent sources to prepare a calibration curve (ranging from 1 to 1000 µg/L) and further, two quality controls within the calibration range. Acceptance criteria were ±15% deviation from the nominal concentrations for calibrators and ±20% for quality controls. Samples were analysed in duplicate, one ‘neat’ and one sample diluted (1 + 9, v/v). In the event of disagreement, the sample was reanalysed for confirmation.

### Calculation of anticipated striatal D_2_ occupancy

Clinical effects of antipsychotics on dopamine D_2_ receptors in the brain have been studied most extensively in the striatum,^24^ which is thought to be of central importance to psychotic phenomena. The Michaelis–Menten equation, used to calculate receptor occupancy from a given drug concentration, is:

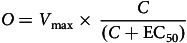

where *O* is occupancy, *C* is concentration, *V*_max_ is maximal potential receptor occupancy and EC_50_ is the concentration resulting in half of maximal potential receptor occupancy. Individual Michaelis–Menten equations for each drug were derived from existing positron emission tomography scan literature.^4,16^ These were used to anticipate striatal D_2_ occupancy from drug concentrations at each research session; *V*_max_ for both flupentixol and zuclopenthixol was assumed to be 100%. The EC_50_ values used were 0.680 µg/L for flupentixol^16^ and 1.158 µg/L for zuclopenthixol.^4^

### Variables

RODOC (percentage points per 30 days) was used as the main predictor variable, while total PANSS score difference between sessions (points at minimal concentration minus those at maximal) was used as the outcome variable. Other predictor variables were separately analysed throughout this study, including absolute difference in D_2_ occupancy during the inter-dose interval (percentage points), predicted D_2_ receptor occupancy at minimal concentration (%) and the duration between sampling sessions (days). Potential confounding variables of age, gender, ethnicity and individual drug were analysed to observe any notable trends within the overall sample, with the aim of controlling to allow examination of the central relationship between RODOC and mental state. Statistical analysis was performed using Stata software (version 18 for Windows; StataCorp LLC, Texas, USA; www.stata.com). We report effect sizes along with 95% confidence intervals, and set the statistical significance level to 0.05.

## Results

### Recruitment and feasibility

**Fig. 1:**
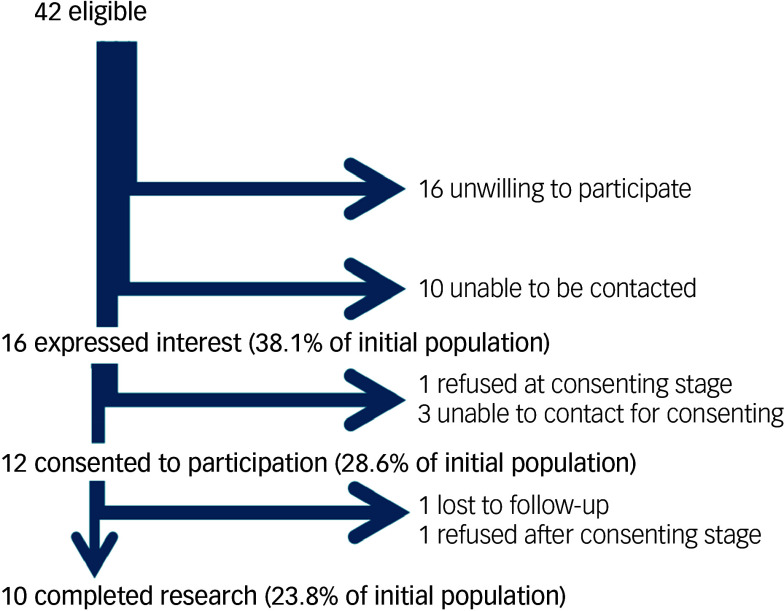
Flowchart demonstrating recruitment and participation during the MIDILIA pilot.

Ten participants were recruited and completed both sampling sessions within the pilot phase of MIDILIA. This represented a recruitment rate of 23.8% from an eligible population of 42 people within the single mental health trust. Figure 1 portrays a flowchart representing the feasibility of recruitment and participation during this study.

### Demographics

Table 1 shows the demographics of our recruited sample. The mean per-weekly dosage of flupentixol LIA in our sample was 43 mg/week (range 5–150 mg/week), whereas the mean per-weekly dosage of zuclopenthixol was 319 mg/week (range 175–600 mg/week). Mean maximal and minimal plasma concentrations for flupentixol were 5.8 and 3.4 µg/L, respectively, while mean maximal and minimal plasma concentrations for zuclopenthixol were 79.0 and 32.6 µg/L, respectively.

**Table 1:** Demographics of recruited sample

Demographics	Overall sample
Gender, *n* (%)	
Male	8 (80)
Female	2 (20)
Age, years, mean (s.d.)	42.5 (10.3)
Ethnicity, *n* (%)	
White British	7 (70)
Black African	3 (30)
LIA drug, *n* (%)	
Flupentixol decanoate	6 (60)
Zuclopenthixol decanoate	4 (40)
Setting, *n* (%)	
Out-patient	5 (50)
In-patient, rehabilitation ward	3 (30)
In-patient, assessment and treatment ward	2 (20)
LIA inter-dose interval, days, *n* (%)	
7	1 (10)
14	3 (30)
21	4 (40)
28	2 (20)
PANSS score, median (IQR), points	
Maximal drug concentration	56 (46–68)
Minimal drug concentration	56 (47–70)

LIA, long-acting injectable antipsychotic; PANSS, Positive and Negative Syndrome Scale; IQR, interquartile interval.

### Mental state differences between maximal and minimal concentration

Global PANSS scores at maximal and minimal drug concentrations were compared and analysed through within-subject *t*-tests. There was no overall significant difference in global mental state between maximal and minimal plasma concentrations during the inter-dose interval. The mean difference between sessions was a mildly increased total PANSS score of 1.3 points at minimal concentration compared with maximal (95% CI [−3.130, 5.730], *P* = 0.524).

**Fig. 2:**
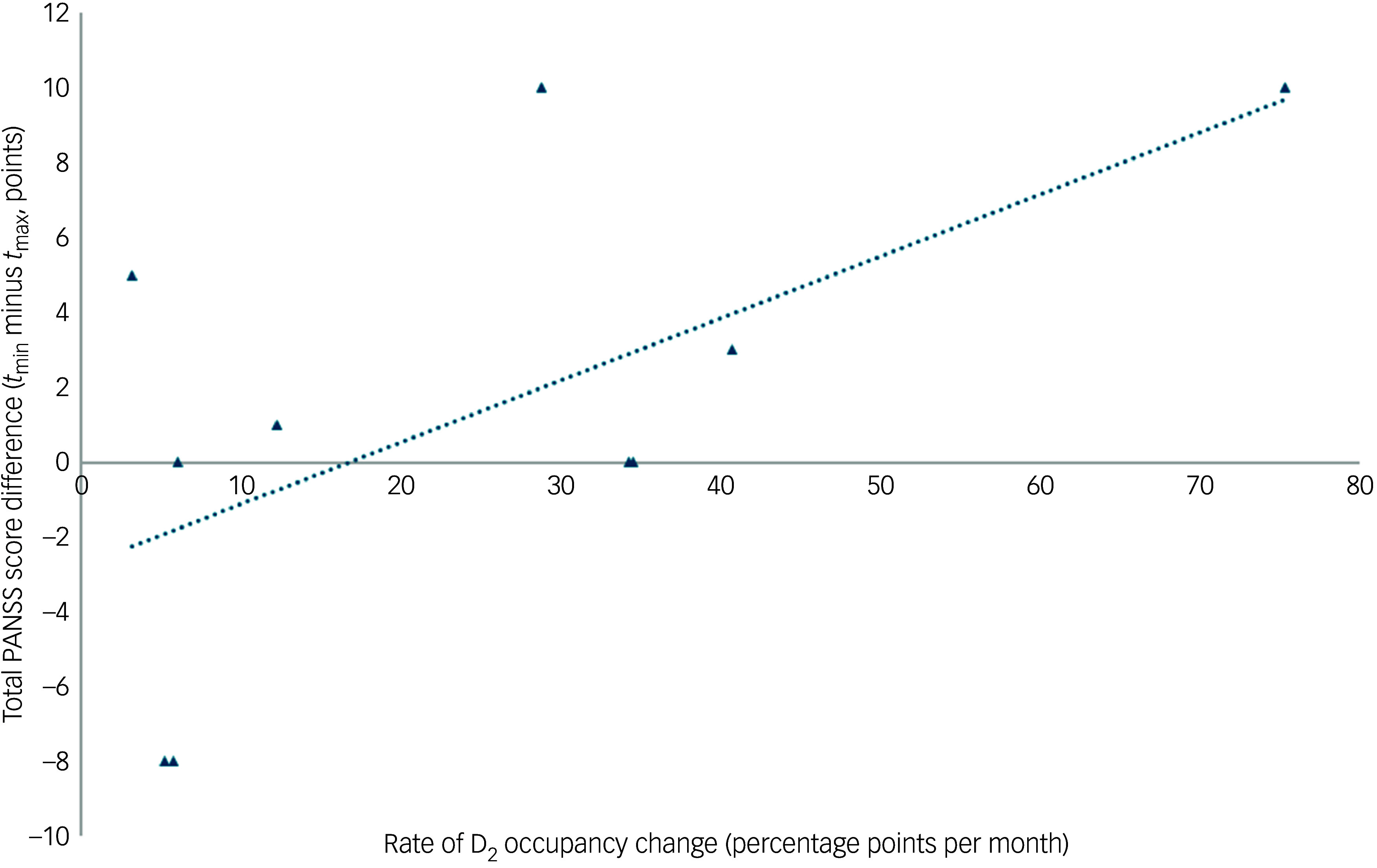
Graph depicting relationship between rate of D_2_ occupancy change and difference in total Positive and Negative Syndrome Scale (PANSS) score between sessions. Difference in PANSS score is calculated by that at the time of minimal drug concentration (*t*_min_) subtracted from that at the time of maximal drug concentration (*t*_max_).

However, a positive trend was observed between RODOC and total PANSS score difference between sampling sessions; more rapid RODOC resulted in increased PANSS scores at minimal drug concentrations; this is demonstrated in Fig. 2.

The effect of RODOC on inter-dose interval mental state change was assessed using simple linear regression, to test whether RODOC was able to predict differences in total PANSS score. The fitted regression model used to predict the difference in total PANSS score at minimal concentration compared with maximal concentration (*Y*) was:

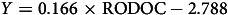




However, analysis demonstrated this regression to be non-significant (*R* ^2^ = 0.376, *P* = 0.059); 95% confidence intervals for the regression coefficient were [−0.008 to 0.340].

In addition, RODOC was categorised into ‘fast’ (>10 percentage point change in D_2_ occupancy per 30 days) and ‘slow’ (<10 percentage point change in D_2_ occupancy per 30 days) groups. A between-subjects *t*-test was applied to compare differences in outcome variable between the two groupings of RODOC. The fast group demonstrated higher scores at minimal concentrations by 4 points (95% CI [−1.010, 9.010]), whereas the slow group had a lower total PANSS score at minimal concentrations by 2.75 points (95% CI [−12.920, 7.421]). The difference between fast and slow groups did not reach statistical significance (95% CI [−1.348, 14.848], *P* = 0.091).

### Exploration of other potential predictor variables

Univariate regression analysis found all potential predictor variables outlined in our methodology to be non-significant in predicting differences in total PANSS score between sessions (Table 2).

**Table 2:** Results of univariate regression sensitivity analyses outlining how various independent variables predicted differences in total Positive and Negative Syndrome Scale score between minimal and maximal drug concentrations

Predictor variable	*R* ^2^	Regression coefficient [95% CI]	*P*-value
Rate of D_2_ occupancy change	0.376	0.166 [−0.008, 0.340]	0.059
Total change in D_2_ occupancy (percentage points)	0.248	0.24 [−0.101, 0.580]	0.143
Minimal D_2_ occupancy (%)	0.198	−0.106 [−0.281, 0.068]	0.198
Time between sessions, days	0.095	0.323 [−0.490, 1.136]	0.387

### Differences between domains on PANSS

**Fig. 3:**
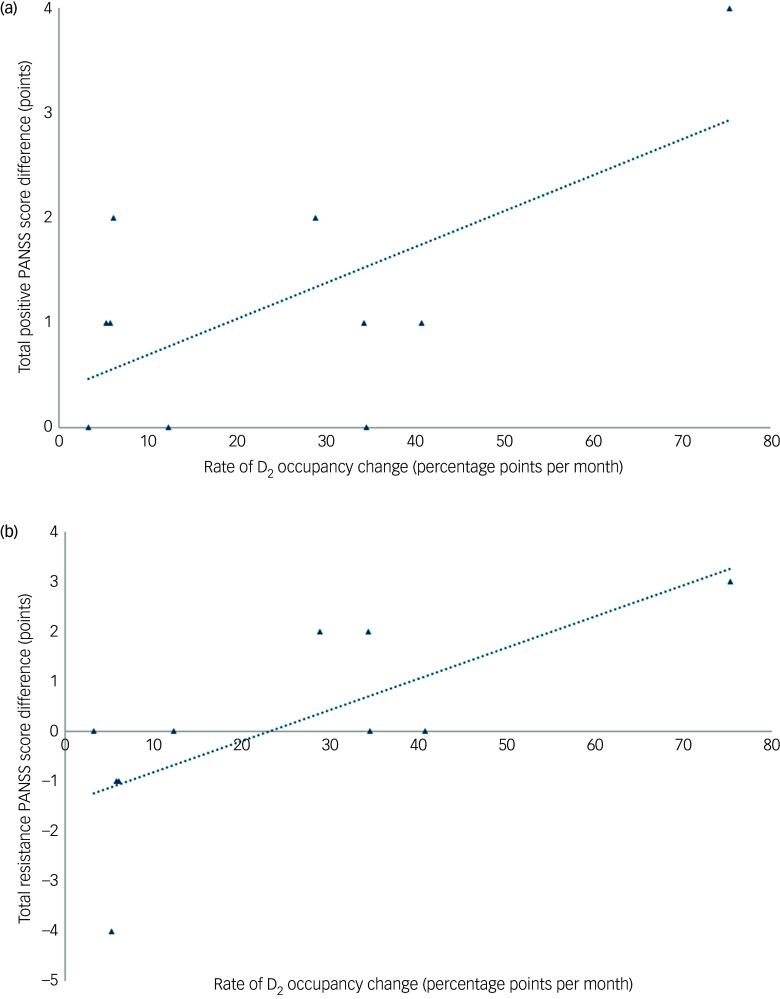
Graphs depicting relationship between rate of D_2_ occupancy change and difference in Positive and Negative Syndrome Scale (PANSS) score between sessions, relating to (a) positive domain and (b) resistance domain. Difference in PANSS score is calculated by that at the time of minimal drug concentration (*t*_min_) subtracted from that at the time of maximal drug concentration (*t*_max_).

Serial univariate regression analysis was also used to compare the role of RODOC as a predictor variable on each of the individual PANSS domains outlined in the five-factor^19^ reclassification. This was utilised to demonstrate whether antipsychotic withdrawal was more prevalent in any specific domain. RODOC significantly predicted increases in scores under the ‘positive’ and ‘resistance’ domains of PANSS as participants approached their minimal drug concentration. There was no significant association between RODOC and the three other PANSS domains. This is further presented in Table 3 and Fig. 3, below.

**Table 3:** Results of univariate regression analyses of how D_2_ receptor occupancy change predicted differences in each of the five domains^19^ on the Positive and Negative Syndrome Scale (PANSS) interview between minimal and maximal drug concentrations

PANSS domain	*R* ^2^	Regression coefficient [95% CI]	*P*-value
Total	0.378	0.166 [−0.008, 0.340]	0.059
Positive	0.406	0.034 [0.001, 0.068]	0.047^*^
Negative	0.001	−0.006 [−0.146, 0.135]	0.928
Disorganisation	0.122	0.037 [−0.044, 0.119]	0.322
Affective	0.144	0.038 [−0.037, 0.113]	0.279
Resistance	0.527	0.062 [0.014, 0.111]	0.018^*^

* Statistical significance with an *α*-value of 0.05.

### Alternative predictor variables to RODOC

Further univariate regression analysis^25^ was used to compare the relationship between total PANSS difference between maximal and minimal concentrations with other potential predictor variables listed in Method, above. Total D_2_ occupancy change during the inter-dose interval also significantly predicted differences in resistance PANSS scores (*R* ^2^ = 0.445, *P* = 0.035), but did not predict differences in positive PANSS scores (*R* ^2^ = 0.249, *P* = 0.142). D_2_ occupancy at minimal drug concentration did not significantly predict differences in PANSS scores for either positive (*R* ^2^ = 0.101, *P* = 0.370) or resistance domains (*R* ^2^ = 0.264, *P* = 0.129).

### Controlling for confounding variables

Potential confounding variables of gender, ethnicity and LIA drug were planned to be analysed through between-subject analysis of variance, with difference in total PANSS score as the outcome variable. However, because only two groups were recruited within each variable, statistical analysis was modified to a between-subject *t*-test. The potential for age as a confounding variable was explored by comparison with difference in total PANSS score through simple linear regression. No trends in total PANSS score differences were observed between difference in age (*R* ^2^ = 0.006, *P* = 0.835), groups of different gender (*P* = 0.363), drug type (*P* = 0.486) or ethnicity (*P* = 0.193).

## Discussion

Our preliminary results suggest that the rate of D_2_ occupancy change (RODOC) may be able to predict deterioration in mental state with regard to positive symptoms of psychosis – for example, delusional thoughts and hallucinations (positive PANSS subscale^19^), as well as scores on hostility, uncooperativeness, impulse control and excitement (resistance PANSS subscale^19^). This could have implications for dose reduction in clinical practice, and may guide clinicians on the early warning signs that may emerge during a tapering regimen to suggest that dose reduction is progressing too rapidly. However, the small sample recruited to date through this pilot study means that these results must be replicated before final conclusions can be made and clinical guidance amended.

### Differences in mental state within the inter-dose interval

Overall, mental state did not significantly fluctuate between administrations of LIA. This may depend, rather, upon the rate at which occupancy of dopamine receptors changes during the inter-dose interval. At low doses and drug concentrations, more pronounced changes in mental state appeared to have taken place during the inter-dose interval, in line with anticipated receptor occupancy changes being of a greater magnitude.

At higher antipsychotic doses and drug concentrations, dopamine receptors are often excessively saturated to the point that large reductions in plasma concentration may not lead to a correspondingly large reduction in receptor occupancy.^16,26^ We found that, for higher doses of LIA (and therefore predicted oversaturation of D2 receptors), mental state was observed to be worse at peak concentrations compared with that at trough concentrations. In other words, participant mental state improved as drug concentration reduced when high doses were used and oversaturation of receptors alleviated. The converse was seen at lower doses of LIA. Five participants in our study had predicted D_2_ occupancies at both maximal and minimal concentration exceeding 95%. The average difference in total PANSS score within this subsample was a reduction of 2 points at minimal concentration compared with maximal. This is in line with previous findings of worse patient outcomes from exceeding ∼80% D_2_ occupancy.^4,5,27^

### Dose reductions for LIAs

Determination of how to prevent the occurrence of symptoms of antipsychotic withdrawal is crucial to devising tolerable tapering regimens to reduce drug dosage. If psychotic symptoms provoked as a result of withdrawal represent a major limitation to dose reduction, it is envisaged that limiting RODOC to a tolerable degree will improve the successful achievement of dose reductions and, potentially in some cases, full discontinuation of psychosis drugs.

Proportionate dose reductions have proven successful in randomised controlled trials for antipsychotics,^28^ benzodiazepines^29^ and opioids.^30^ Hyperbolic tapering has also been observed to be successful for people taking antidepressants.^31,32^ However, implementing dose reduction regimens that accommodate linear reductions in receptor occupancy is challenging in regard to LIAs, given their infrequent dosing and fluctuance in drug concentration during inter-dose intervals. Previous in silico modelling by some of this study’s authors has recommended that a switch to oral medication may be required at some stage during a reduction in LIA dose.^10,11^ However, empirical evidence is currently lacking in terms of the specific point during a taper that this should take place.

### Evidence for a minimally effective D_2_ occupancy for antipsychotic efficacy

The intention of multiple univariate linear regression analyses through MIDILIA was to establish whether RODOC is in fact the optimal predictor variable in anticipating potential deterioration in mental state. An alternative consideration was whether a minimally effective and therapeutic D_2_ occupancy existed, and whether reduction below this would result in the emergence of symptoms of psychosis. The latter has often been referenced with respect to an ‘optimal D_2_ receptor range’ being between 65 and 80%.^27,33^

From our preliminary results, we found no evidence that reduction in antipsychotic drug plasma concentrations below that expected to correspond with a D_2_ occupancy of 65% resulted in increased deterioration in mental state. Rather, it appears that faster RODOC more closely predicted the emergence of both positive and resistance symptoms on PANSS interviews. More rapid RODOC is observed at lower doses of psychosis drugs, which may explain why this concept of a minimally effective receptor occupancy has been postulated. This will be an area of further focus as we expand the study with a larger sample.

### Strengths and limitations

The methodology of MIDILIA has proven to be a practicable and feasible approach to observing how people tolerate variation in drug concentration during the inter-dose interval of LIAs. Our recruitment rate and feasibility calculations suggest that our preliminary findings can be confirmed through modest expansion of our geographical catchment and the inclusion of other psychosis drugs in our recruitment strategy.

Observational studies are often subject to greater influence from confounding variables when compared with controlled interventional trials. Therefore, the former are often considered to carry less evidential weight in terms of academic literature.^34^ However, MIDILIA capitalises on an opportunity to explore the naturalistic variation of psychosis drugs, providing novel and informative findings without altering clinical practice.

Previous antipsychotic discontinuation trials have found increased rates of relapse with reduced dosage in the short term,^35^ thus leading to a reduction in some clinicians’ confidence in considering and recommending dose reduction to their patients. Observational studies will therefore play an important role before further dose reduction trials can, and should, be conducted. This is certainly the case for MIDILIA, whereby the required rate for reduction in terms of D_2_ receptor reduction can be assessed before testing through a clinical trial.

MIDILIA did not incorporate researcher blinding in terms of when sampling sessions took place during the inter-dose interval. This could be considered a limitation in terms of distinguishing mental state between maximal and minimal concentrations, and could be addressed in a replicated study with greater resources provided to facilitate this. However, the main predictor variable during the study was RODOC between the two examination points. Both researcher and participant remained blinded with regard to group assignment at the point of the research being conducted, due to the nature of each participant’s plasma concentration not being known until after interviews had been conducted. This is due to individual metabolism and pharmacokinetic factors not being known until serum assay results had been obtained. As an example of the degree to which these factors can effect drug concentration, flupentixol has been found to have a sixfold variation in plasma concentration for the same dose.^9^ This highlights the considerable differences that may occur between different participants, and which would be detected only once serum assays had been analysed.

Due to the design of MIDILIA observing participants only during their inter-dose interval, the longer-term effects and successes of reducing the dose of antipsychotic, further than minimal plasma concentrations observed during the study, cannot be determined. Therefore, conclusions relating to this cannot be made on the basis of this study design alone, and will be determined only through future interventional work. Previous discontinuation trials have reported poorer patient outcomes in the short term as a result of rapid and linear dose reductions.^35^ However, longer-term studies of psychosis drug dose reductions have demonstrated improved outcomes.^36^ It is important that observational work such as MIDILIA is first conducted to elucidate the required rate at which any proposed interventional dose reduction should occur, to avoid adverse outcomes from tapering in the short term.

Finally, it is noted that there was a lack of even representation of participants across gender and ethnicity within our study. Our sample predominantly recruited White British males and, while this may be explained in part by the higher incidence of schizophrenia among males,^37^ psychotic disorders are reported to be considerably more prevalent within ethnic minority groups.^38^ The disparity between the epidemiology of psychosis and our sample is one that we aim to address as we expand our recruitment through sampling frames and more targeted recruitment. However, preliminary results suggest that difference in gender or ethnicity would not affect the relationship between RODOC and mental state changes.

### Implications for further research and clinical practice

We are planning to continue with the MIDILIA study over the coming years through expansion of our inclusion criteria, both geographically to incorporate other NHS mental health trusts as well as recruiting participants taking other LIAs of haloperidol,^39^ risperidone and paliperidone.^16,40^ The methodology employed has, to date, proven to be pragmatic and feasible, and we hope to demonstrate statistically significant results that support our preliminary indicative findings.

An equality sample size calculation, with a significance level of 5% and power set at 80%, was used to determine the sample size required to achieve a statistically significant finding regarding the relationship between RODOC and global mental state changes. This utilised the differences between the groups of either fast or slow RODOC, as described in the Method section above. Sample size calculation indicated the required sample size to be 28 participants. Using the recruitment rate of 23.8% from this pilot, it is envisaged that a total population of 118 people would need to be approached in order to recruit our required sample.

The aim for clinicians treating psychosis should be to establish optimised dosing for LIAs (i.e. as low a dose as possible) that would result in an unchanged mental state throughout the inter-dose interval, and between minimal and maximal drug concentrations. Our preliminary results suggest that an approximate 15 percentage point change in striatal D_2_ occupancy per 30 days did not lead to any changes in mental state during the inter-dose interval of LIAs. Whether this rate of change would be tolerable if continued throughout a taper to full discontinuation remains unclear, due to the short-term nature of this study.

MIDILIA was an observational study, and therefore could analyse only existing clinical practice. However, findings from this may be used alongside reanalysis of existing trial data^28,35^ to compare successes between different tapering regimens employed in interventional trials. Once there is further clarity around the required tolerable rate of antipsychotic reduction from observational study, further interventional research will be required to test dose reductions of LIAs in a hyperbolic manner.

In conclusion, MIDILIA has demonstrated its practicability and feasibility through a successful pilot phase. Preliminary findings suggest that changes in mental state may be more likely to manifest as a faster rate of D_2_ receptor occupancy change, particularly with respect to positive symptoms of psychosis, as well as hostility, impulse control, excitement and co-cooperativeness.^19^ However, further recruitment is required before statistical and clinical significance can be achieved.

## Supporting information

O’Neill et al. supplementary material O’Neill et al. supplementary material

## Data Availability

The data that support the findings of this study are available from the corresponding author, J.R.O., upon reasonable request.

## Supplementary material

The supplementary material is available online at https://doi.org/10.1192/bjo.2025.10831
