# Supplementary material for: Correlations between receptor occupancy change and mental state in patients using long-acting injectable antipsychotics: MIDILIA pilot study
Source: BJPsych Open. 2025 Sep 12;11(5):e211. doi: 10.1192/bjo.2025.10831 (PMC12451532; doi:10.1192/bjo.2025.10831)
Supplement: O’Neill et al. supplementary material [file S2056472425108314sup001.docx]

***Supplementary Material 1: Graphical presentations of individual participant predicted D_2_ occupancy and total PANSS score at minimal and maximal drug concentrations during the inter-dose interval. Blue line represents predicted D_2_ occupancy, and orange line represents total PANSS score.***
